# Disruption of fibronectin fibrillogenesis affects intraocular pressure (IOP) in BALB/cJ mice

**DOI:** 10.1371/journal.pone.0237932

**Published:** 2020-08-21

**Authors:** Jennifer A. Faralli, Mark S. Filla, Colleen M. McDowell, Donna M. Peters

**Affiliations:** 1 Department of Pathology and Laboratory Medicine, School of Medicine and Public Health, University of Wisconsin, Madison, Wisconsin, United States of America; 2 Department of Ophthalmology and Visual Sciences, School of Medicine and Public Health, University of Wisconsin, Madison, Wisconsin, United States of America; University of Iowa, UNITED STATES

## Abstract

Increased deposition of fibronectin fibrils containing EDA+fibronectin by TGFβ2 is thought to be involved in the reduction of aqueous humor outflow across the trabecular meshwork (TM) of the eye and the elevation in intraocular pressure (IOP) observed in primary open angle glaucoma (POAG). Using a fibronectin-binding peptide called FUD that can disrupt fibronectin fibrillogenesis, we examined if disrupting fibronectin fibrillogenesis would affect IOP in the TGFβ2 BALB/cJ mouse model of ocular hypertension. BALB/cJ mice that had been intravitreally injected with an adenovirus (Ad5) expressing a bioactive TGFβ2^226/228^ showed a significant increase in IOP after 2 weeks. When 1μM FUD was injected intracamerally into mice 2 weeks post Ad5-TGFβ2 injection, FUD significantly reduced IOP after 2 days. Neither mutated FUD (mFUD) nor PBS had any effect on IOP. Four days after FUD was injected, IOP returned to pre-FUD injection levels. In the absence of TGFβ2, intracameral injection of FUD had no effect on IOP. Western blotting of mouse anterior segments expressing TGFβ2 showed that FUD decreased fibronectin levels 2 days after intracameral injection (p<0.05) but not 7 days compared to eyes injected with PBS. mFUD injection had no significant effect on fibronectin levels at any time point. Immunofluorescence microscopy studies in human TM (HTM) cells showed that treatment with 2ng/ml TGFβ2 increased the amount of EDA+ and EDB+ fibronectin incorporated into fibrils and 2μM FUD decreased both EDA+ and EDB+ fibronectin in fibrils. An on-cell western assay validated this and showed that FUD caused a 67% reduction in deoxycholate insoluble fibronectin fibrils in the presence of TGFβ2. FUD also caused a 43% reduction in fibronectin fibrillogenesis in the absence of TGFβ2 while mFUD had no effect. These studies suggest that targeting the assembly of fibronectin fibrillogenesis may represent a way to control IOP.

## Introduction

Glaucoma is a group of eye diseases that causes irreversible damage to the optic nerve leading to vision loss and blindness. Elevated intraocular pressure (IOP) is the most common risk factor for developing a glaucoma subtype called primary open angle glaucoma (POAG). The elevation in IOP is thought to arise from an increase in resistance to aqueous humor outflow across the trabecular meshwork (TM) which is due in part to an excessive increase in extracellular matrix (ECM) proteins and a decrease in ECM turnover in the TM [1–3]. The increase in ECM may be due to the elevated levels of TGFβ2 found in the aqueous humor of many POAG patients [4–8]

TGFβ2 has been reported to increase both IOP and secretion of fibronectin [9] in human anterior segment organ cultures and decrease outflow facility [10]. Both TGFβ1 and TGFβ2 also induced an increase in IOP with a correlated increase in fibronectin labeling in the TM in a rat model [11, 12]. Among the ECM proteins upregulated by TGFβ2 in human TM (HTM) cells is fibronectin, including the EDA+ and EDB+ isoforms of fibronectin [13–15].

Fibronectin fibrils are a major component of the ECM for many cells, including TM cells [16–18] and its assembly into fibrils by TM cells has been shown to regulate the deposition of other ECM components including laminin, fibrillin, and type IV collagen [19]. Other ECM proteins dependent upon fibronectin for their assembly into fibrils include collagen types I and III [20–24]. As in other tissues, fibronectin fibrillogenesis by TM cells is a highly regulated cell surface receptor-mediated stepwise process involving numerous intermolecular interactions that is controlled by the contractile properties of a tissue [25–28] and integrin signaling [25, 29–31]. While α5β1 integrin is the main integrin involved in fibronectin fibrillogenesis, other integrins such as αvβ3 integrin can also participate in fibrillogenesis [32, 33].

Although fibronectin fibrillogenesis is dependent on a number of different intermolecular interactions during fibril formation [34–39], the 70 kilodalton (kDa) fragment from the amino terminus of fibronectin is critical for this process [40]. Hence, peptides or small molecules that target this domain in fibronectin can prevent the assembly of fibronectin into fibrils. One such peptide is called FUD and is derived from the functional upstream domain (FUD) of the *Streptococcus pyogenes* adhesin F1 protein [41]. FUD (or pUR4) can inhibit fibronectin matrix assembly by fibroblasts by binding to the amino-terminal 70kDa fragment of fibronectin [42–46]. FUD has also been shown to inhibit the deposition of fibronectin in experimental fibrosis in the liver [46], in vascular remodeling [47] and in a murine model of fibrosis in the kidney [45].

A recent study showed that FUD can prevent *de novo* fibronectin fibril formation in both untreated and in dexamethasone treated cultures of HTM cells where fibronectin fibrillogenesis is normally upregulated *in vitro* [19, 25] and during ocular hypertension *in vivo* [48]. In addition, FUD was able to indirectly prevent the incorporation of type IV collagen, laminin and fibrillin into newly forming matrices, but did not have any effect on these proteins once they had been incorporated into a mature matrix [19]. Interestingly, FUD treated HTM cell cultures did show a loss of fibronectin that had been deposited pre-FUD treatment, suggesting that the prevention of fibronectin fibrils can lead to the turnover of pre-existing fibronectin matrices. This suggests that targeting the amino terminus of fibronectin may be a potential treatment to prevent the increase in fibronectin deposition observed in POAG.

In this study, we determined if FUD could prevent the increase in fibronectin matrix assembly and deposition seen in TGFβ2 treated HTM cell cultures. We also tested if FUD affects IOP in the Ad5-TGFβ2 mouse model of ocular hypertension [49] which exhibits an increase in fibronectin expression. These studies showed that FUD prevented fibronectin fibrillogenesis in response to TGFβ2 and promoted the removal of pre-existing fibronectin fibrils in mature matrices *in vitro*. Intracameral injections of FUD also transiently lowered IOP and partially decreased fibronectin levels in the Ad5-TGFβ2 mouse model.

## Materials and methods

### Materials

Rabbit polyclonal anti-fibronectin sera was produced in our lab and verified by ELISA using purified plasma fibronectin and by immunofluorescence microscopy [19, 50]. Mouse anti-succinate dehydrogenase complex flavoprotein subunit A (SDHA; ab14715), mouse Ist-9 (ab6328) and mouse BC-1 (ab154210) antibodies were obtained from Abcam (Cambridge, MA). Mouse anti-glial fibrillary acidic protein (GFAP) antibody clone GA5 was purchased from Millipore Sigma (G3893, St. Louis, MO). Normal rabbit serum was purchased from Vector Laboratories (Burlingame, CA). Recombinant FUD or a mutated recombinant FUD (mFUD) with reduced fibronectin binding was expressed in *E*. *coli* and purified as previously described [19, 51]. For some experiments, FUD was tagged with Alexa 488 (Thermo Fisher Scientific, Waltham, MA) as previously described [19]. Recombinant human TGFβ2 was obtained from R&D Systems (Minneapolis, MN). An adenoviral vector expressing a bioactivated TGFβ2^226/228^ transgene (Ad5-TGFβ2) was purchased from the University of Iowa Gene Transfer Vector Core or supplied by Dr. Abbott Clark (University of North Texas Health Science Center, Fort Worth, TX). The Ad5.null virus was purchased from the University of Iowa Gene Transfer Vector Core.

### Animal studies

All animal studies were carried out in accordance with the Association for Research in Vision and Ophthalmology Statement for the Use of Animals in Ophthalmic and Vision Research and were approved by the Institutional Animal Care and Use Committee of the University of Wisconsin-Madison School of Medicine and Public Health (protocol # M005242). Male BALB/cJ or C57BL/6J mice were ordered from Jackson Laboratory (Bar Harbor, ME) and were housed in the University of Wisconsin animal facilities with a 12-hour light/12-hour dark cycle with food and water freely available. All experiments were conducted with mice after 6 weeks of age to allow the TM to fully develop [52].

To determine if FUD could affect IOP, BALB/cJ mice were injected intravitreally with an Ad5-TGFβ2^226/228^ virus as previously described [49]. Briefly, mice were first anesthetized intraperitoneally with a ketamine/xylazine mix (90mg/10mg per kg). Anesthetized mice were then given topical 0.5% proparacaine (Alcon; Fort Worth, TX) to numb the eye. A 30g needle was then used to poke a hole in the eye posterior to the limbus. The Ad5-CMV-TGFβ2^226/228^ virus or Ad5.null virus containing no transgene was intravitreally injected using 1.5μl of 5x10^10^ plaque forming units/ml with a 10μl Hamilton syringe and a 33g beveled needle (World Precision Instruments, Sarasota, FL). Fifteen days after injection, some mice were injected intracamerally with FUD, mFUD or PBS. For this, mice were anesthetized with a ketamine/xylazine mix as described above. Topical 0.5% proparacaine was then applied to numb the eye and 3μl of 1μM FUD, 1μM mFUD or PBS was injected intracamerally into the eye as previously described [53]. At the end of the experiments, mice were euthanized and the eyes were enucleated and processed for paraffin embedding or bisected and anterior segments were lysed for western blotting.

In some experiments, C57BL/6J mice received intracameral injection of 1μM Alexa 488-conjugated FUD in order to determine where FUD localized in the anterior segment. For this, mice were anesthetized with a ketamine/xylazine mix as described above. Topical 0.5% proparacaine was then applied to numb the eye. Three microliters of 1μM Alexa 488-conjugated FUD solution was injected intracamerally into one eye of C57BL/6J mice as previously described [53]. Four hours or 3 days after the injection, mice were euthanized and the enucleated eyes were bisected. The anterior segment was flattened by making four radial cuts into the segment and mosaic images where then acquired with an epifluorescence microscope (Axioplan 2, Carl Zeiss Microscopy, LLC, White Plains, NY) equipped with a digital camera (Axiocam HRm; Carl Zeiss Microscopy, LLC) and image acquisition software (Axiovision v. 4.8; Carl Zeiss Microscopy, LLC).

### Western blot analysis

Dissected mouse anterior segments were immediately placed in 200μl ice cold lysis buffer (25mM Hepes, pH 7.4, 150mM NaCl, 1mM EDTA, 1mM NaF, 1% NP-40, 0.25% deoxycholic acid (DOC), and Halt^TM^ protease and phosphatase inhibitor cocktails (Thermo Fisher Scientific)). Tissue was then sonicated (Branson Sonifier SLPe, Thermo Fisher Scientific) 5 times for 1 second using 30% amplitude. The insoluble material was removed by centrifugation at 14,000 rpm for 10 min at 4˚C. A Micro BCA Protein Assay Kit (Thermo Fisher Scientific Pierce) was used to determine protein concentration in the supernatant. Proteins in the supernatant (10μg) were separated on a 4–20% SDS-PAGE gel (Bio-Rad, Hercules, CA) and transferred to Immobilon-FL (Millipore Sigma; Burlington, MA). The membrane was blocked with 3% BSA in Tris buffered saline with 0.5% Tween-20 (TBST) for at least 1 hour at room temperature. Membranes were then incubated overnight at 4˚C with rabbit anti-fibronectin sera (1:1000) or mouse anti-SDHA (1:5000) diluted in 1% BSA in TBST. After washing, membranes were incubated for 1 hour at room temperature with IR Dye 800 conjugated goat anti-mouse or anti-rabbit secondary antibody (LI-COR Biosciences, Lincoln, NE) diluted 1:15,000 in 1% BSA in TBST with 0.01% SDS. Membranes were washed and then digitally scanned (Odyssey CLx imager, LI-COR). Densitometry of the bands was determined using Image Studio v. 5.0 software (LI-COR). The density of the fibronectin bands was normalized to the density of the bands of the housekeeping gene SDHA within each sample.

### IOP measurements

**Fig 1:**
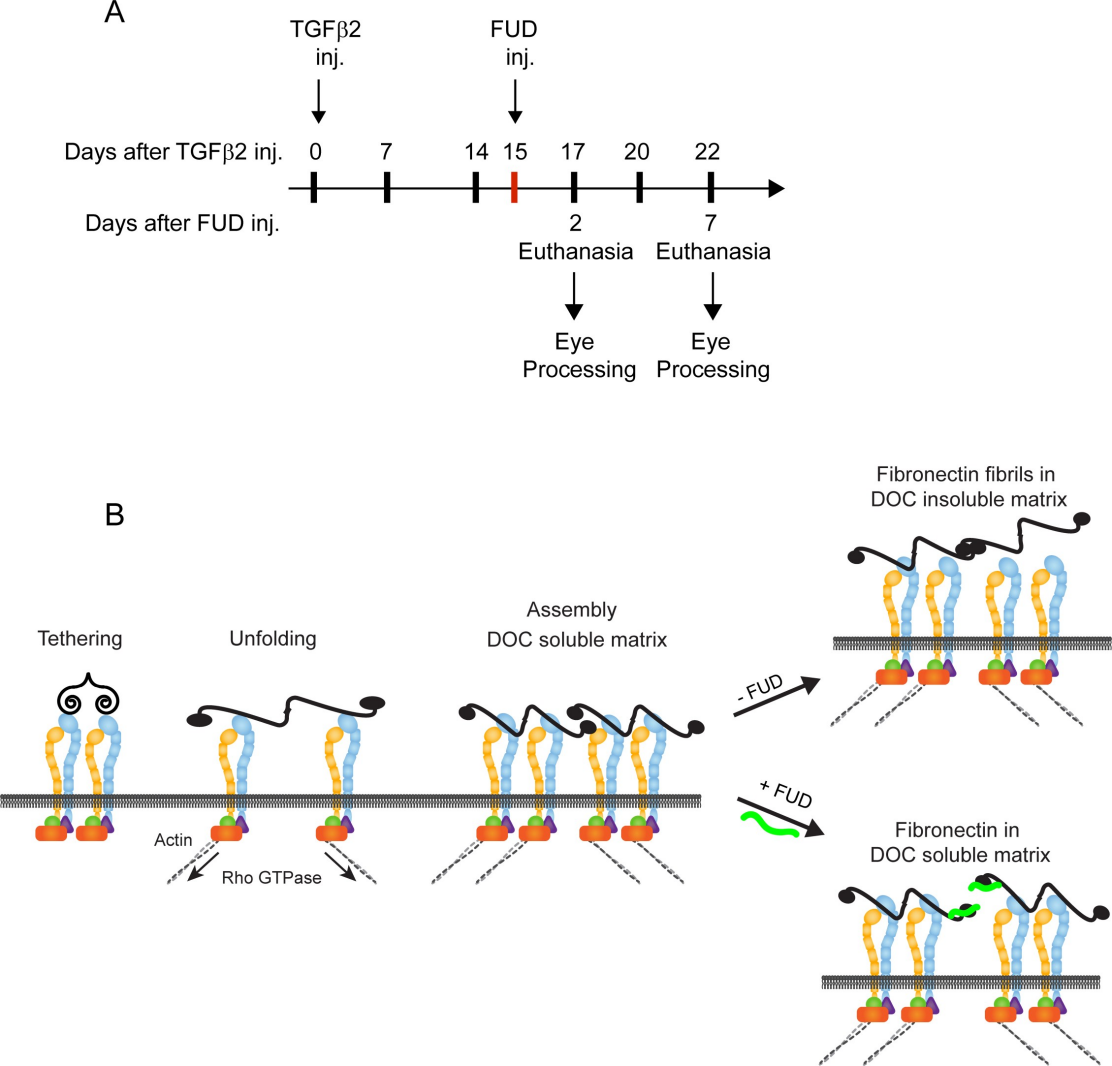
Treatment timeline and model for fibronectin matrix assembly. (A) Timeline showing which days IOP measurements were taken (black hash marks) and when Ad5-TGFβ2 (Day 0) or FUD (Day 15; red hash mark) was injected into the eye. Inj. = day injection was done. (B) The assembly of fibronectin into fibrils starts when a globular fibronectin dimer binds an integrin on the cell surface. Contractile forces applied from the actomyosin cytoskeleton via the integrins causes the fibronectin dimer to unfold, exposing fibronectin-fibronectin binding sites that mediate fibril formation. Additional fibronectin dimers are incorporated into the extracellular matrix creating a deoxycholate (DOC) insoluble fibril. FUD (green line) inhibits the additional incorporation of fibronectin into the extracellular matrix by blocking fibronectin-fibronectin binding sites necessary for fibril formation.

Mice (7–10 weeks of age) were anesthetized intraperitoneally with a ketamine/xylazine mix (90mg/10mg per kg). IOP was measured within 2 minutes after the mice no longer responded to a toe pinch and before the anesthesia effect on IOP occurred using a rodent Icare Tonolab (Raleigh, NC) [54, 55]. IOPs were measured by the same person between 10-11am to avoid differences due to circadian rhythms. Three IOP measurements from each eye were averaged together at each time point. Fig 1A indicates when IOP was measured. Baseline IOP was established prior to any treatments.

### Immunohistochemistry

Whole mouse eyes were fixed and embedded in paraffin as previously described [53]. Sagittal sections 5μm thick were cut and stained with hematoxylin 3333 and eosin to assess the morphology of the trabecular meshwork. Images were obtained using a brightfield microscope (Olympus Corp.; New Orleans, LA).

### Cell culture

HTM cells were isolated and expanded from donor eyes of a 17 year old male, two different 27 year old females and a 25 year old male as previously described [56–58] and designated as N17TM-2, N27TM-2, N27TM-6 and N25TM-8 respectively. All eyes had no history of ocular disease. Cells were characterized using established procedures [56–59] and grown in low glucose DMEM (Sigma, St. Louis, MO), 15% fetal bovine serum (FBS; Atlanta Biologicals, Atlanta, GA), 2 mM L-glutamine (Sigma), 1% amphotericin B (Mediatech, Herndon, VA), 0.05% gentamicin (Mediatech) and 1ng/ml FGF-2 (Peprotech Rocky Hill, NJ). Cells were grown to confluency and were used in experiments one week later.

### Immunofluorescent microscopy

HTM (N25TM-8) cells were plated on glass coverslips and grown to confluency. One week later cells were treated for 4 days with 2μM FUD with or without 2ng/ml TGFβ2 in media containing reduced serum (1%) and no FGF-2. Media was replaced every other day with FUD with or without TGFβ2. After 4 days, cells were fixed with -20˚C methanol for 15 minutes, incubated with a blocking buffer (PBS plus 1% BSA) and then double labeled with anti-fibronectin sera (1:800) and Ist-9 antibody (5μg/ml) or BC-1 antibody (5μg/ml) overnight at 4˚C in blocking buffer. Normal rabbit serum (1:800) and anti-GFAP antibody (5μg/ml) were used as negative controls. Primary antibodies were detected with Alexa 488 goat anti-rabbit or Alexa 546 goat anti-mouse conjugated secondary antibodies (1:500; Thermo Fisher Scientific). Nuclei were labeled with Hoechst 33342. Fluorescence was viewed using an epifluorescence microscope (Axioplan 2; Carl Zeiss Microscopy, LLC) equipped with a digital camera (Axiocam HRm; Carl Zeiss Microscopy LLC) and image acquisition software (Axiovision ver. 4.8, Carl Zeiss Microscopy LLC). All images within an experiment were processed in the exact same way by changing the brightness or contrast to optimize the images.

### On-cell western (OCW) analysis

HTM cells (N17TM-2, N27TM-2 and N27TM-6) were plated into 96 well plates at 15,000 cells per well. One week after the cells reached confluency media was replaced with media containing 1% FBS and 2ng/ml TGFβ2 with and without 2μM FUD. Two days later the cells were processed for OCW analysis as previously described [19]. Briefly, cells were extracted with a hypotonic buffer (20mM HEPES, pH 7.4, 1mM EDTA, 1% sodium deoxycholate and HALT^TM^ protease inhibitor (Thermo Fisher Scientific)) leaving behind insoluble extracellular matrix (ECM). The DOC insoluble ECM was fixed with 4% paraformaldehyde and the total protein in the wells was labeled with IRDye 680 NHS ester (LI-COR). Wells were then blocked and fibronectin was detected using rabbit anti-fibronectin sera followed by the IRDye 800CW-conjugated goat anti-rabbit IgG (LI-COR). The plates were then read on a LI-COR Odyssey CLx scanner and analyzed using the LI-COR Image Studio v. 5.0.21 software. The antibody signal was normalized to the corresponding NHS ester signal (total protein).

### Data analysis

Data are presented as mean ± standard error of the mean (S.E.M). Statistical comparisons were done using the unpaired Student t-test (GraphPad QuickCalcs) or one-way ANOVA together with the post-hoc Tukey HSD test (OCW). A p value <0.05 was considered significant.

## Results

### FUD affects TGFβ2-induced increase in IOP *in vivo*

To determine if the formation of fibronectin fibrils affected IOP *in vivo*, fibronectin fibrillogenesis was induced in BALB/cJ mice using a one-time intravitreal injection of an adenovirus Ad5-TGFβ2^226/228^ vector that expresses a bioactive TGFβ2 transgene as previously described [49]. Fifteen days later, as shown in the timeline in Fig 1A, the FUD peptide was intracamerally injected to prevent any further assembly of fibronectin into fibrils in the presence of TGFβ2. As shown in Fig 1B, FUD, which binds to the amino terminal type I repeats of fibronectin [19, 25, 42, 46], inhibits the incorporation of soluble cell surface bound fibronectin into insoluble fibrils.

**Fig 2:**
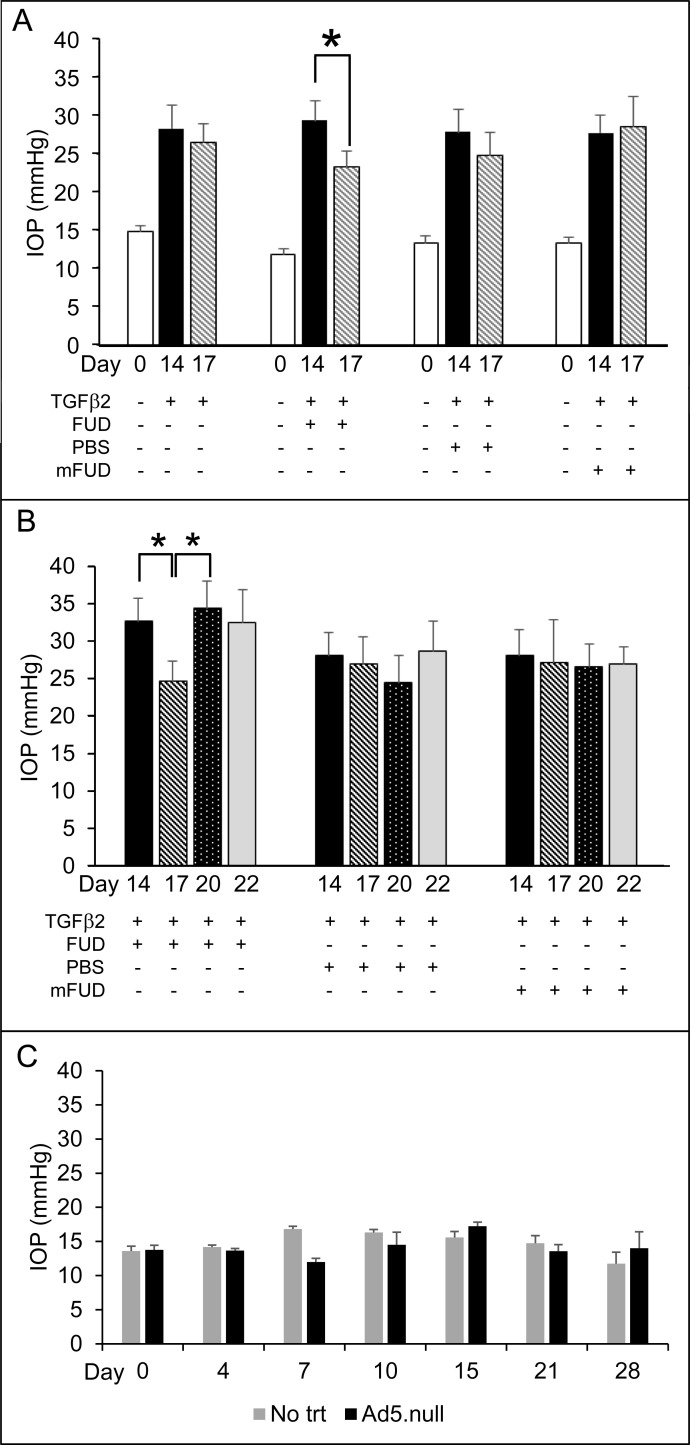
FUD transiently decreased IOP in mice injected with Ad5-TGFβ2 viral vector. (A) Graph shows IOPs measured on Days 0, 14 and 17 in mice injected with TGFβ2 adenovirus. On Day 15, some TGFβ2 transduced mice received intracameral injections of FUD, PBS or mFUD. FUD significantly decreased IOP on Day 17 (2 days after it was injected on Day 15; *p<0.05). n = 6, 15, 13 and 12 for TGFβ2 only, TGFβ2 + FUD, TGFβ2 + PBS and TGFβ2 + mFUD treated eyes respectively. (B) IOPs were monitored for 5 more days (until Day 22) in a subset of mice from A. Graph shows IOPs measured on Days 14, 17, 20 and 22 in mice injected with Ad5-TGFβ2 viral vector. On Day 15, some TGFβ2 transduced mice received intracameral injections of FUD, PBS or mFUD. FUD significantly decreased IOP on Day 17 (2 days after FUD injection) (*p<0.05). IOP then returned to baseline pre-FUD injection levels on Day 20 (5 days after injection). n = 10, 9 and 8 for TGFβ2 + FUD, TGFβ2 + PBS and TGFβ2 + mFUD treated eyes respectively. (C) Graph shows that injection of the Ad5.null viral vector containing no transgene had no effect on IOP (n = 4). No trt = contralateral eye that was uninjected. All graphs show mean ± SEM of IOPs at that time point. Statistical analysis was performed using an unpaired Student t-test.

Fig 2A shows that mice receiving the bioactive TGFβ2^226/228^ transgene showed an elevation in IOP by day 14 following the injection, similar to what has been shown by others [49]. When FUD was injected intracamerally into the mouse eye with the elevated IOP, we saw a 20% decrease in IOP of 5.9±1.4mmHg (p<0.05) 2 days after the FUD injection on day 17 (compare 29.2±2.6 mmHg on Day 14 to 23.3±2.0 mmHg on Day 17). In contrast, intracameral injection of a mutated FUD (mFUD) had no significant effect on IOP (27.6±2.4 mmHg on Day 14 compared to 28.5±3.8mmHg on Day 17). However, the effect of FUD on IOP was transient. As shown in Fig 2B, the IOP of the mice receiving FUD returned to pre-FUD injection levels on day 20 (compare 32.7±3.1 mmHg on Day 14 vs. 34.4±3.7 mmHg on Day 20). This increase in IOP to pre-FUD levels remained at day 22. Fig 2C shows injection with Ad5.null virus had no effect on IOP at any time point.

**Fig 3:**
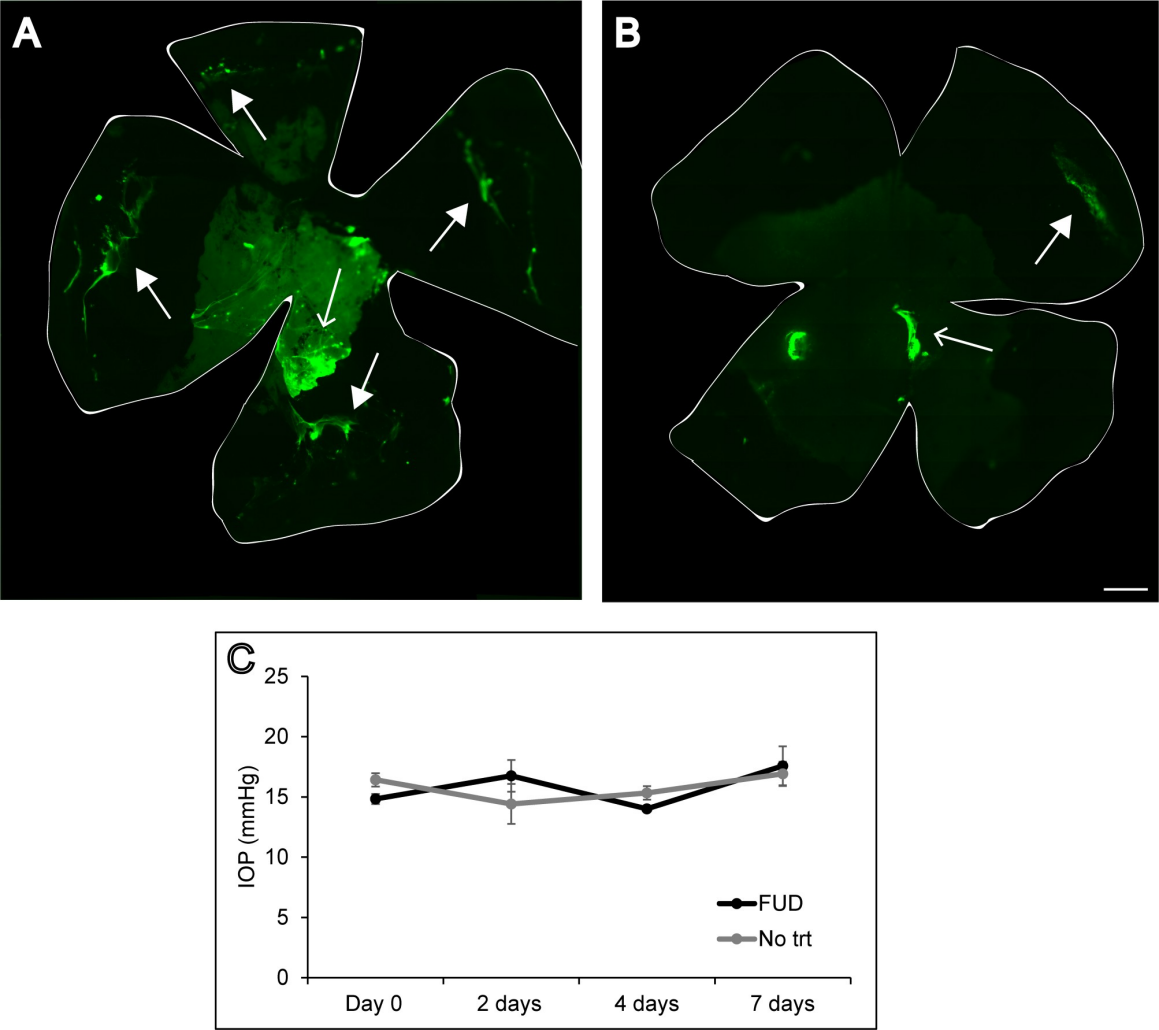
Alexa 488-conjugated FUD effect on IOP and localization in the eye. C57BL/6 mouse anterior segment whole mounts 4hours (A) or 3 days (B) after receiving Alexa 488-conjugated FUD. The perimeter of the whole mounts is indicated by the while lines. Arrows point to Alexa 488-conjugated FUD in the TM. Open arrows point to Alexa 488-conjugated FUD in the cornea. Bar = 500μm. (C) Graph shows IOP of C57BL/6 mice injected intracamerally with 1μM Alexa 488-conjugated FUD to one eye. The contralateral eyes (No trt) did not receive an injection. No points were statistically significant, n = 4 eyes. Data is shown as mean ± SEM of the IOP measured at that time point. Statistical analysis was performed using an unpaired Student t-test.

The subsequent increase in IOP in FUD-treated mice at day 20 (Fig 2B) was most likely due to the wash out of the FUD peptide from the TM. As shown in Fig 3A, Alexa 488-conjugated FUD can be seen in the TM in whole mounts of anterior segments of the mouse eye 4 hours after injection (arrows) and in the cornea, especially at the injection site (open arrows). After 3 days (Fig 3B), only a small amount of FUD can still be found in the TM and at the injection site in the cornea. Fig 3C shows that intracameral injection of Alexa 488-conjugated FUD had no significant effect on IOP over a 7 day period in the absence of TGFβ2 expression.

Interestingly, TGFβ2 transduced eyes that received no intracameral injection or an intracameral injection of PBS (Fig 2A) also showed a slight decrease in IOP at day 17 of 7% (28.2±3.0 mmHg on Day 14 compared to 26.5±2.4 mmHg on Day 17) and 11% (27.8±2.9 mmHg on Day 14 compared to 24.7±3.1 mmHg on Day 17) respectively. This slight decrease was not statistically significant and is probably due to the expression of the Ad5-TGFβ2 transgene starting to decline [49]. This decrease in TGFβ2 expression may be due to the young age of mice used in this study, since studies using older mice (> 5 months) showed a sustained increase in IOP for at least 8 weeks [60].

**Fig 4:**
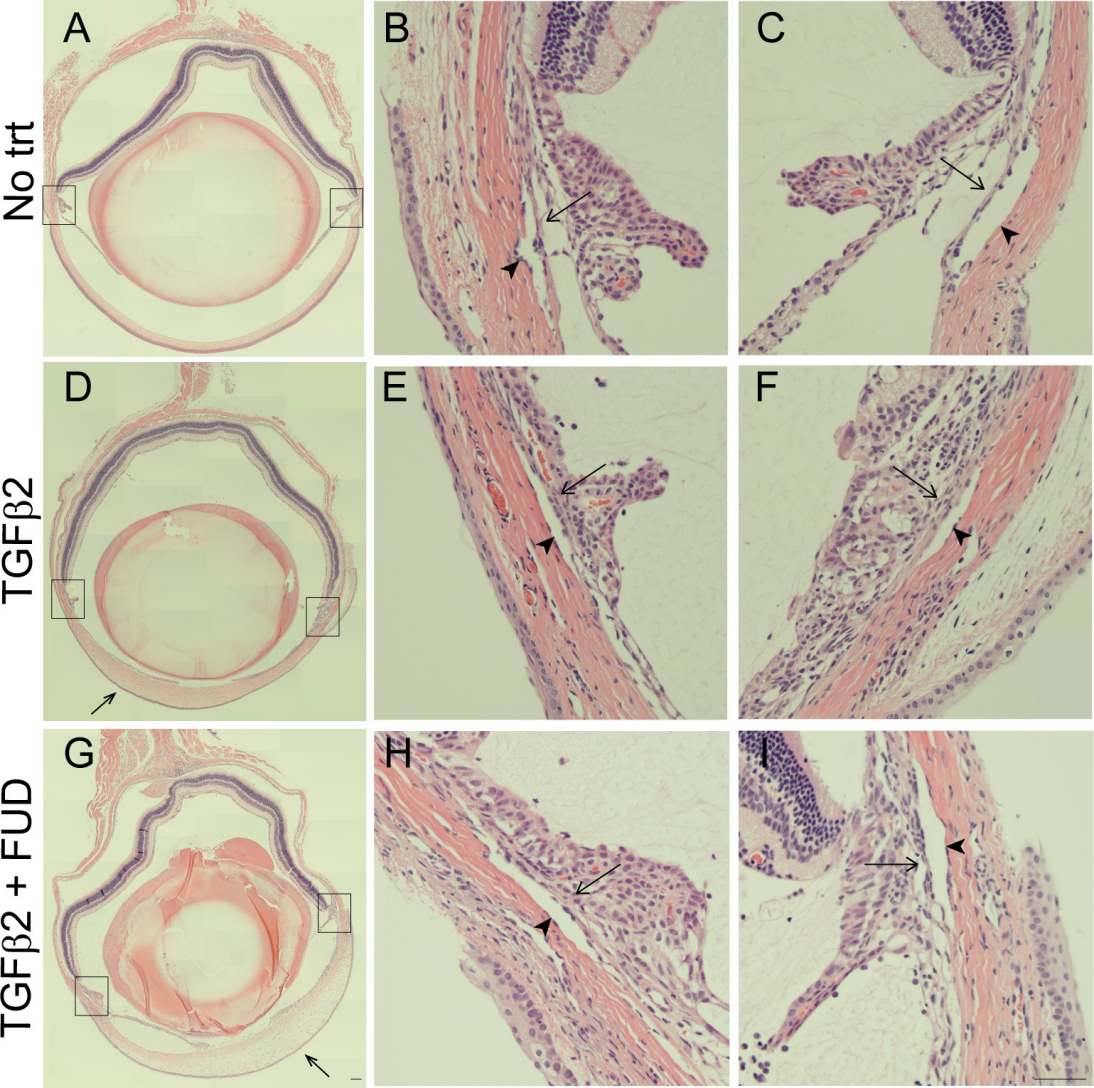
TGFβ2 and FUD treatment affected mouse eye morphology. Hematoxylin and eosin staining of an uninjected (No trt) mouse eye (A), a mouse eye 22 days after an intravitreal injection of Ad5- TGFβ2 viral vector (D) and a mouse eye 22 days after an intravitreal injection of Ad5-TGFβ2 vector with an intracameral FUD injection on Day 15 (G). Higher magnifications of the trabecular meshwork and Schlemm’s Canal of the control eye (B, C), TGFβ2 treated eye (E, F) and TGFβ2 + FUD treated eye (H, I). Micrographs are representative. Boxes in A, D and G are the TM shown in higher magnification in B, C, E, F, H and I. Closed arrows indicate corneal edema, open arrows indicate the TM and arrowheads indicate Schlemm’s Canal. Bar (G) = 100μm, bar (I) = 50μm.

To determine if the injections of TGFβ2 and FUD affected the overall morphology of the anterior segment, some eyes were paraffin embedded and stained with hematoxylin and eosin (H&E). The morphology of the TM appeared unchanged in eyes expressing the TGFβ2 transgene compared to control eyes not expressing TGFβ2 transgene. But as seen in some previous studies, eyes expressing TGFβ2 exhibited a thickening of the cornea (Fig 4D) as well as a partially closed angle where the iris is adhered to the corneal endothelium in parts of the eye compared to a control eye (Fig 4A) [49]. These changes could be due to the age of the animals used in this study since a previous study using older mice did not report these changes [61]. A higher magnification of the trabecular meshwork (Fig 4E and 4F) shows that Schlemm’s canal is intact. Eyes expressing TGFβ2 and subsequently injected with FUD showed a similar pathology of a thickening of the cornea and a closed angle (Fig 4G) as eyes expressing the TGFβ2 transgene (Fig 4D).

### Changes in fibronectin levels after FUD injection

**Fig 5:**
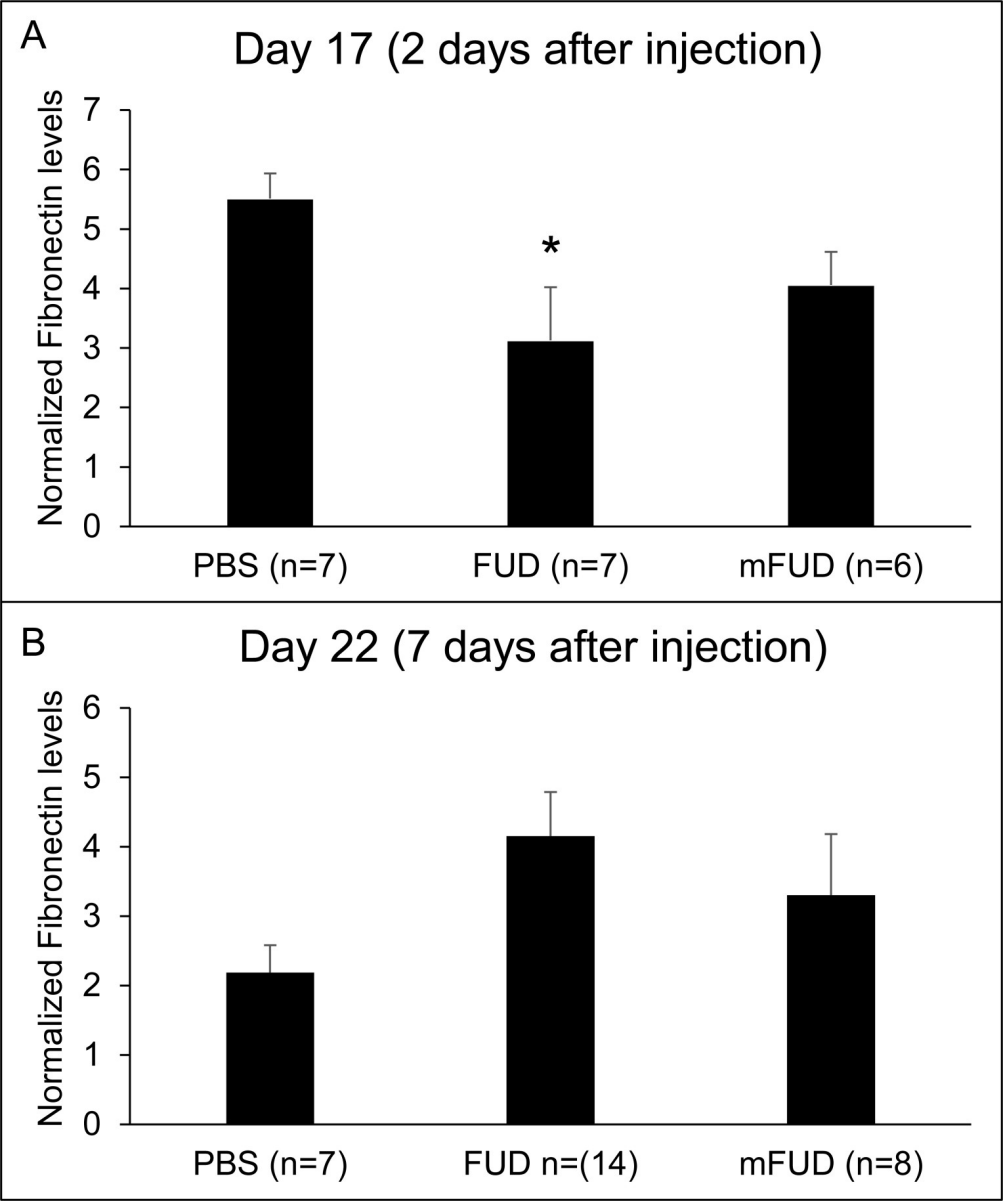
FUD lowered fibronectin levels in the TGFβ2 transduced mouse eyes. (A) Western blot densitometry analyses of lysates from TGFβ2 transgene expressing mice treated for 2 days (Day 17) with PBS, FUD or mFUD probed with anti-fibronectin sera. The same western blots were then probed with anti-SDHA antibody as a housekeeping gene. The density of the fibronectin bands were normalized to SDHA for each sample. Data are the mean ± SEM of the normalized fibronectin bands for each treatment group. Fibronectin levels in TGFβ2 + PBS treated eyes were significantly different from levels in TGFβ2 + FUD treated eyes (*p<0.05). Statistical analysis was performed using an unpaired Student t-test. (B) Western blot densitometry analyses of lysates, probed with anti-fibronectin sera, from TGFβ2 transduced mice treated for 7 days with PBS, FUD or mFUD. The same western blots were then probed with anti-SDHA antibody as a housekeeping gene. The density of the fibronectin bands was normalized to SDHA for each sample. Data are the mean ± SEM of the normalized fibronectin bands for each treatment group. There were no statistically significant differences between the data points. Statistical analysis was performed using an unpaired Student t-test.

To determine if FUD decreases the amount of fibronectin *in vivo*, we performed western blot analyses using anterior segments from mice expressing the bioactive TGFβ2 and treated with FUD, PBS or mFUD for 2 days (17 days post TGFβ2 injection) or 7 days (22 days post TGFβ2 injection) (see IOP data above; Fig 2). Anterior segments for these experiments contained the cornea, iris, trabecular meshwork, ciliary muscle and sclera. As shown in Fig 5A and S1 Fig on day 17, 2 days after the FUD, PBS or mFUD injections, fibronectin levels were 43% lower in eyes injected with FUD compared to eyes injected with PBS (p < 0.05). Fibronectin levels were also 26% lower in mFUD treated eyes compared to PBS treated eyes, but this was not a significant decrease (p = 0.4). This suggests that the presence of FUD led to lower levels of fibronectin in the presence of TGFβ2. The effect of mFUD is not unexpected since mFUD retains some partial, although not statistically significant, activity in HTM cultures [19]. A similar observation was made in fibroblasts using a deletion mutant of FUD [42]. By day 22, 7 days post intracameral injection of FUD, when IOP levels are up again, there is no difference in fibronectin levels in TGFβ2 treated eyes that received FUD, PBS or mFUD intracameral injections (Fig 5B and S2 Fig).

### FUD disrupts TGFβ2 induced fibronectin fibrillogenesis in HTM cultures

**Fig 6:**
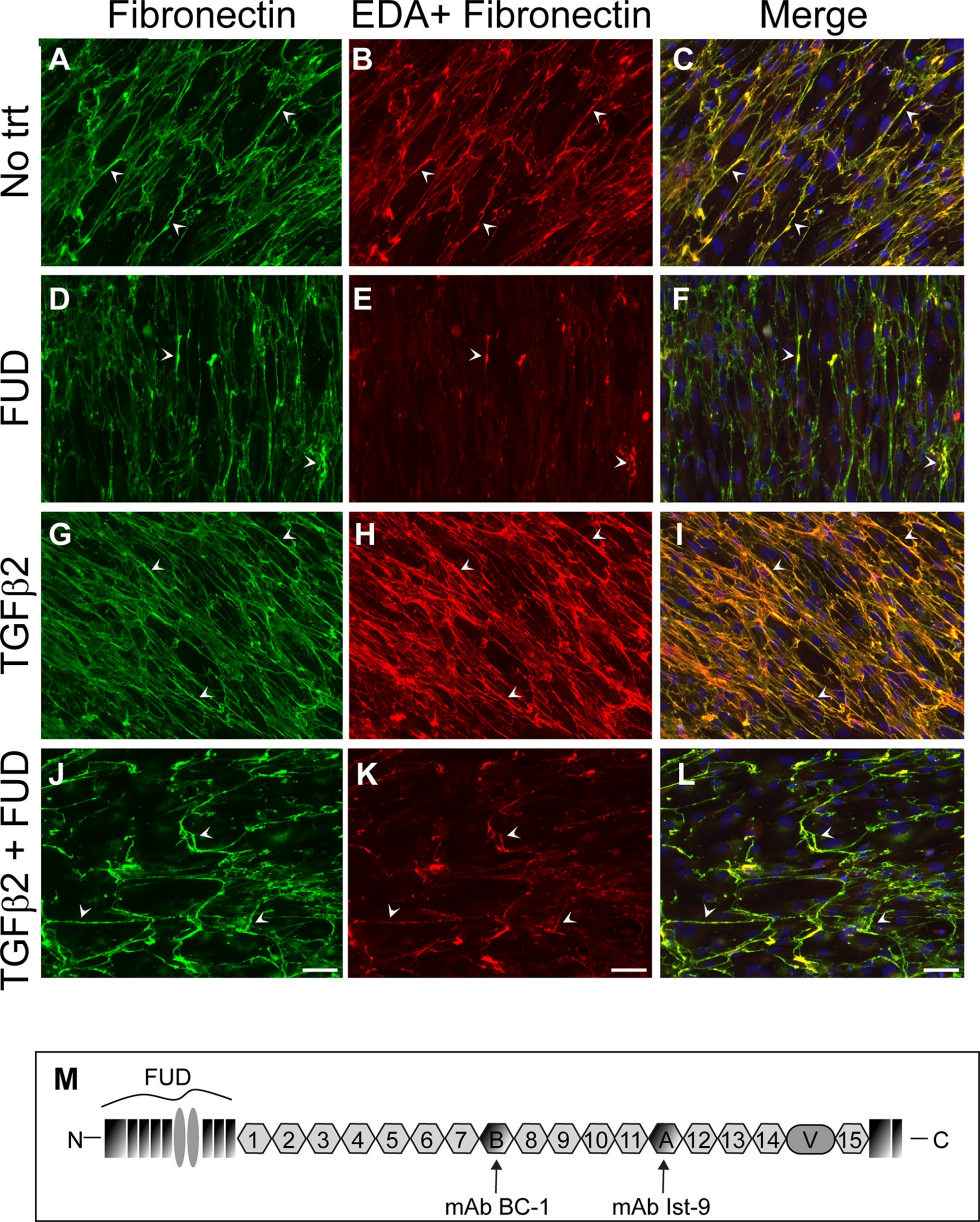
Recombinant FUD prevented TGFβ2 induced increase in fibronectin and EDA+ fibronectin fibrillogenesis. Confluent HTM cultures (N25TM-8) were untreated (A-C) or treated with 2μM FUD (D-F), 2ng/ml TGFβ2 (G-I) or both FUD and TGFβ2 (J-L) for 4 days. Cells were then fixed and double labeled with a polyclonal fibronectin sera (A, D, G and J) or an antibody (Ist-9) that recognizes the EDA domain of fibronectin (B, E, H and K). Arrow heads in merged fibronectin and EDA+ fibronectin images (C, F, I and L) indicated where fibronectin and EDA+ fibronectin labeling coincide. Similar results were seen with one other cell strain. Bar = 50μm. (I) Schematic of fibronectin indicates that FUD binds the N-terminal end of fibronectin and location of the epitopes recognized by the monoclonal antibodies Ist-9 and BC-1.

**Fig 7:**
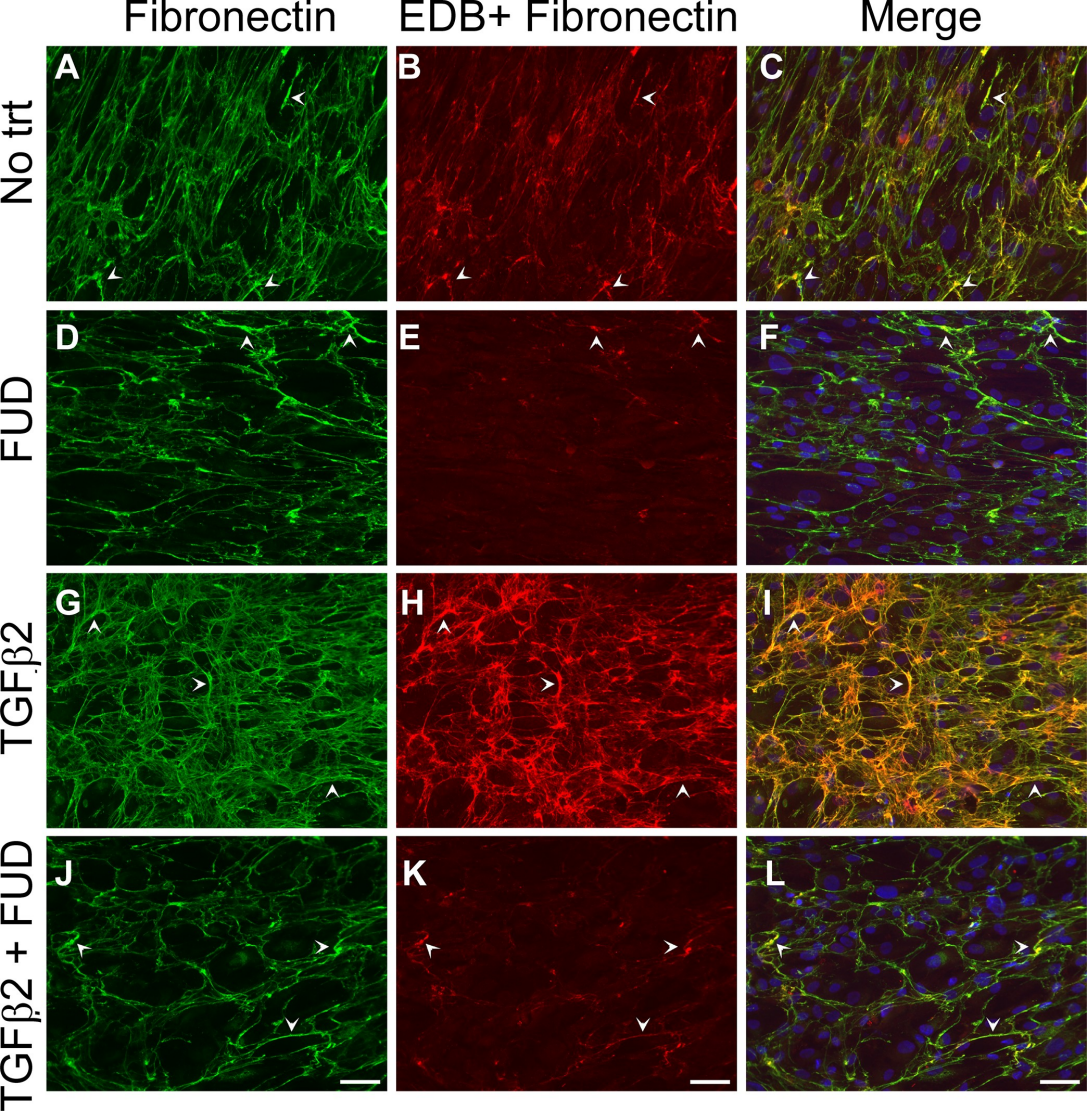
Recombinant FUD prevented TGFβ2 induced increase in fibronectin and EDB+ fibronectin fibrillogenesis. Confluent HTM cultures (N25TM-8) were untreated (A-C) or treated with 2μM FUD (D-F), 2ng/ml TGFβ2 (G-I) or both FUD and TGFβ2 (J-L) for 4 days. Cells were then fixed and double labeled with polyclonal fibronectin sera (FN, A, D, G and J) or an antibody (BC-1) that recognizes the EDB domain of fibronectin (B, E, H and K). Arrowheads in merged fibronectin and EDB+ fibronectin images (C, F, I and L) indicate where fibronectin and EDB+ fibronectin labeling coincide. Similar results were seen with one other cell strain. Bar = 50μm.

Since it is difficult to quantify fibronectin fibrillogenesis *in vivo* (See Fig 1), we turned to *in vitro* studies using TGFβ2 treated HTM cultures. As shown in Figs 6 and 7, HTM cultures expressed both the EDA+ and EDB+ isoforms of fibronectin by immunofluorescence microscopy. As seen in previous immunofluorescence microscopy studies [19], confluent HTM cells form a fibronectin matrix around the periphery of the cells (Figs 6A and 7A). Most of the fibronectin made by HTM cells contained the EDA+ isoform of fibronectin (Fig 6B and merged in C). HTM cells also expressed the EDB+ fibronectin isoform, although in lesser amounts (Fig 7B and merged in C). Treating cells with TGFβ2 increased the levels of both EDA+ and EDB+ fibronectin in HTM cultures (Figs 6H and 7H) compared to cultures not treated with TGFβ2.

Treating HTM cultures with FUD prevented the formation of fibronectin fibrils within the matrix. FUD decreased the amount of total fibronectin (Figs 6D and 7D) and EDA+ and EDB+ fibronectin fibrils (6E and 7E, respectively) in HTM cultures in the absence of TGFβ2. FUD also prevented the formation of fibronectin (Figs 6J and 7J) and EDA+ and EDB+ fibronectin fibrils in TGFβ2 treated cultures (Figs 6K and 7K respectively). No labeling was seen using nonimmune rabbit sera or anti-GFAP as controls (S3 Fig). Taken together, these results show that FUD prevents the assembly of soluble fibronectin into fibrils in both untreated and TGFβ2 treated HTM cells.

**Fig 8:**
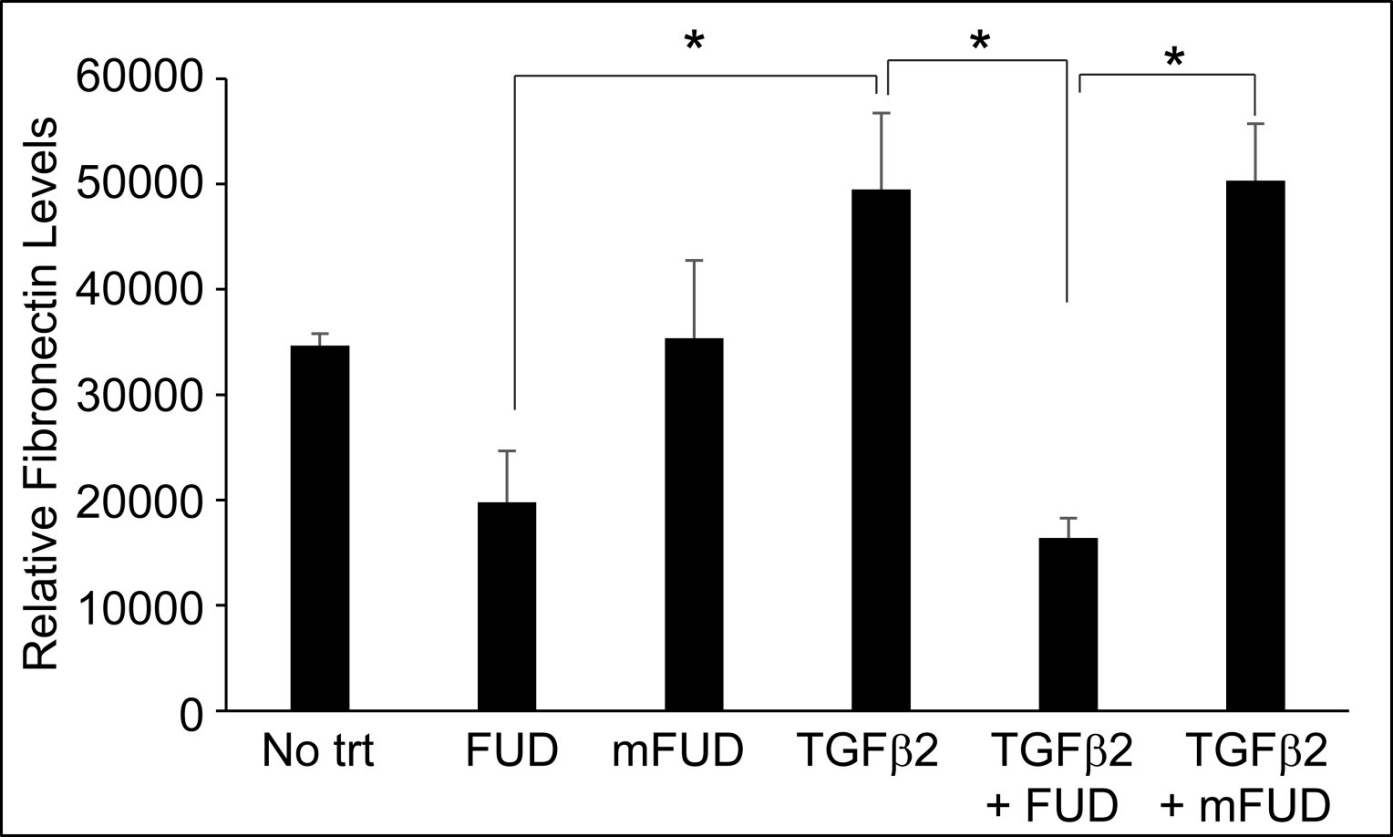
On cell western (OCW) showed FUD prevented the TGFβ2 induced increase in DOC-insoluble fibronectin fibrils. HTM cells were plated into 96 well plates. One week after reaching confluency cells were treated with or without 2μM FUD or mFUD, 5 ng/ml TGFβ2, FUD + TGFβ2 or mFUD + TGFβ2. After 2 days cell layers were extracted with 1% DOC then processed for OCW analysis. Data is the average of 3 cell strains performed in duplicate (N17TM-2, N27TM-2, and N27TM-6) ± SEM. No trt = cells not treated with TGFβ2, FUD or mFUD. Statistically significantly different by one-way ANOVA with the post-hoc Tukey test (*p<0.05).

To further demonstrate that FUD can prevent fibronectin fibrillogenesis, we quantified fibronectin fibril formation by first extracting soluble fibronectin from the cell layers with deoxycholate (DOC) and then quantifying the remaining insoluble, crosslinked fibronectin fibrils using an on-cell western technique [19, 25]. Fig 8 shows that TGFβ2 treatment increased the amount of insoluble fibronectin fibrils by 42% (p<0.01) compared to no treatment and FUD was able to inhibit this increase. FUD significantly decreased the amount of fibrils by 43% compared to no treatment and 67% compared to TGFβ2 treatment (p<0.01). The FUD effect on fibronectin fibrillogenesis was specific, since mFUD had no statistically significant effect on fibronectin fibrillogenesis in the absence or presence of TGFβ2.

## Discussion

In this study we used an inhibitory peptide called FUD that specifically prevents fibronectin fibrillogenesis to explore the role of fibronectin fibrils in TGFβ2-induced ocular hypertension. Our studies show that disrupting fibronectin fibrillogenesis using the FUD peptide caused a decrease in the TGFβ2-induced elevation in IOP seen in mice. In both *in vitro* and *in vivo* analyses, FUD reduced the formation of fibronectin fibrils in HTM cultures and the deposition of fibronectin fibrils in mouse anterior segments in the presence of TGFβ2. Taken together, these data suggest that fibronectin fibrils can play a pivotal role in TGFβ2-induced IOP elevation and inhibiting fibronectin fibril incorporation into the ECM may be one way to control IOP.

The temporary IOP lowering effect of FUD in the mouse model was not unexpected because FUD was administered as a one-time injection. FUD is a small peptide fragment of only 49 amino acids. It is likely that FUD washed out of the TM as there was little FUD still present in the anterior segment whole mounts 3 days post-FUD injection. Thus, it is unlikely that FUD would have a longer effect on IOP in the anterior segment. Longer term studies using additional FUD injections could not be done as the mouse cornea cannot tolerate multiple injections without compromising its integrity.

While we saw a significant decrease in fibronectin by western blot analysis of TGFβ2 expressing mouse anterior segments treated with FUD for 2 days, fibronectin was not completely removed as it was in the tissue culture studies. This is most likely due to the fact that the TGFβ2–induced increase in fibronectin deposition had been going on for over 2 weeks prior to the treatment with FUD. If we had administered FUD at the same time we injected the eye with the Ad5-TGFβ2 vector, we would have expected to see a greater difference similar to what we saw in the HTM cell cultures, since we would have prevented the *de novo* assembly of fibronectin into fibrils induced by TGFβ2. We chose not to administer the FUD and Ad5-TGFβ2 vector at the same time because we were more interested to see if we could cause a decrease in fibronectin levels once those levels had increased rather than a prevention in the elevation of fibronectin levels by TGFβ2. Previous *in vitro* studies, however, suggest that if we could have done a longer term treatment with FUD [19, 25], we might have been able to prevent both the *de novo* fibril formation and the removal of fibrils that had been deposited prior to FUD treatment. However, as we discussed above, those longer term studies were not possible to do in this *in vivo* model.

Another factor that may have prevented us from seeing a greater decrease in fibronectin levels is that intracameral injections induce an inflammatory response [49, 62] followed by a wound repair process and both these processes by themselves trigger an upregulation in fibronectin expression [63–65]. Thus, measuring changes in fibronectin expression 2 to 3 days after an injection may be obscured in part by the inflammatory response/wound healing response. Interesting, more recent studies using mice older than 5 months, show these mice do not respond to Ad5-TGFβ2 viral injection with corneal edema and closed iridocorneal angles [60, 61] suggesting these mice may exhibit a lower inflammatory response to the virus. Thus, using older mice in which the immune response is likely to be reduced [66] may make it easier to see the effect of FUD on fibronectin levels without confounding inflammatory responses.

FUD did not show a preference for one isoform of fibronectin over another in HTM cultures here and in an earlier study [19]. These studies showed that treatment of HTM cells with FUD prevented the increased incorporation of both the EDA+ and EDB+ isoforms of fibronectin into fibrils induced by TGFβ2 [13–15]. Controlling the incorporation of the EDA+ isoform of fibronectin is particularly important. This isoform of fibronectin is upregulated during times of tissue rearrangement such as wound healing or in diseased states such as cardiac hypertrophy, diabetic nephropathy, tumors, and in liver and pulmonary fibrosis where its expression is often associated with fibrosis or an epithelial-mesenchymal transition in adults [67–69]. It is also upregulated in glaucomatous human donor eyes [14] which, along with the increased levels of TGFβ2 in the aqueous humor of POAG patients [4–8], may play a role in the pathogenesis of POAG by controlling the differentiation of TM cells into myofibroblast-like cells and altering the contractile properties of the tissue [70, 71].

Whether FUD can be used as a therapeutic treatment for POAG is an interesting question. FUD has been used to prevent fibrosis in both kidney and liver *in vivo* [45, 46]. It works because FUD prevents the formation of fibronectin fibrils and hence their incorporation into the ECM. FUD prevents fibrosis by blocking the formation of fibronectin fibrils that, in turn, provide a scaffold for the deposition of collagen and other components of the ECM [19–24] that contribute to fibrotic changes in tissues. These studies showed that targeting fibronectin to lower IOP was effective even after IOP and fibronectin levels had been elevated by TGFβ2. However, long term delivery of FUD to the eye would be needed and this could prove to be challenging. In addition, no one really knows the long term effects of preventing fibronectin fibrillogenesis on the function of the ECM.

In summary, this study supports previous studies showing that fibronectin does seem to play a role in regulating IOP. In addition, it presents evidence that inhibiting fibronectin fibrillogenesis in the TM may be a way to control IOP. Clearly, future studies exploring ways to get long-term expression of FUD and finding other ways to control fibronectin fibrillogenesis as well as removing pre-existing fibronectin fibrils is needed.

## Supporting information

S1 Fig Western blots used to generate data shown in Fig 5A. Blots have been spliced together to remove the contralateral untreated eye next to the lanes with treated eyes since these lanes were not used in the analyses. Blots were first incubated with the rabbit anti- fibronectin sera and were not stripped before incubating with the SDHA antibody.
(EPS)

S2 Fig Western blots used to generate data shown in Fig 5B. Blots have been spliced together to remove the contralateral untreated eye next to the lanes with treated eyes since these lanes were not used in the analyses. Blots were first incubated with the rabbit anti-fibronectin sera and were not stripped before incubating with the SDHA antibody. Some blots were incubated with both the anti-fibronectin sera and SDHA antibodies simultaneously.
(EPS)

S3 Fig Control images for Figs 6 and 7. Confluent HTM cultures (N25TM-8) were fixed and double labeled with (A) anti-GFAP antibody shown in green (B) normal rabbit serum (non -immune) shown in red. (C) Merged image of A and B including nuclei labeled with Hoechst 33342 in blue. Bar = 50μm.
(EPS)
